# Electrochemical determination of terbutaline using a SnO_2_ nanoparticle-modified carbon paste electrode

**DOI:** 10.55730/1300-0527.3907

**Published:** 2026-07-15

**Authors:** Rauf GULAMOV, Şenol ALPAT, Sibel KILINÇ ALPAT

**Affiliations:** 1 Department of Biotechnology, The Graduate School of Natural and Applied Sciences, Dokuz Eylul University, İzmir, Turkiye; 2 Division of Chemistry Education, Buca Faculty of Education, Dokuz Eylul University, İzmir, Turkiye

**Keywords:** Nanosensor, terbutaline hemisulfate, beta-2 agonists, differential pulse voltammetry, cyclic voltammetry

## Abstract

Terbutaline hemisulfate (THS), a selective beta-2 agonist, is preferred as a bronchodilator for the treatment of asthma and bronchitis, as well as a tocolytic agent to delay labor in acute situations during premature birth. The use of beta-2 agonists above a certain dose is considered doping in sports competitions. Therefore, it is important that they be determined with high sensitivity and selectivity within a short analysis time. In this study, a SnO_2_ nanoparticle-modified carbon paste electrode was developed for the determination of THS. Cyclic voltammetry and differential pulse voltammetry were employed as electrochemical methods. The operating conditions of the developed SnO_2_ nanoparticle-modified carbon paste electrode (SnO_2_NP/CPE), including the scan rate, SnO_2_ nanoparticle content, and pH, were optimized. At the end of the experiments, the optimum operating conditions were determined to be a scan rate of 10 mV/s, 5% SnO_2_ nanoparticles, and 0.25 M acetate buffer at pH 4.0. The analytical parameters of SnO_2_NP/CPE were also determined. The linear range of the nanosensor was 2.0–50.0 μM, with a limit of detection of 0.55 μM and a limit of quantification of 1.8 μM. In the reproducibility and repeatability tests, the coefficients of variation for 30 μM THS, based on 10 measurements, were 2.58% and 3.30%, respectively. No significant interference from NaCl, glucose, urea, and uric acid was observed in the interference experiments. In contrast, ascorbic acid, dopamine, epinephrine, and citric acid showed slight interference. Comparing the sensor response obtained on the 14th day with the initial peak current value of the nanosensor revealed no change in the nanosensor response, indicating that the developed nanosensor has good storage stability. The developed nanosensor (SnO_2_NP/CPE) can be prepared easily and used directly for the determination of THS in samples without additional separation or extraction steps. It is considered suitable for the determination of THS because of its advantages, including good reproducibility and reliability, low cost, and a short response time.

## 1. Introduction

Beta-2 agonists are the primary class of bronchodilators used for acute symptom relief in asthma management. All selective beta-2 agonists produce potent bronchodilation. They differ in their onset of action and duration of action^1^. Terbutaline and salbutamol are widely used beta-2 agonists in many countries because of their short duration of action and the resulting need for frequent dosing [1]. In some cases, because these drugs are commonly prescribed and readily accessible, they may be subject to overdose. However, beta-2 agonists have been abused for doping purposes by athletes [2]. While certain nonasthmatic athletes may experience enhanced adrenergic stimulation and a potential^2^ anabolic-like effect that improves performance (i.e. increased power or prolonged activity), asthmatic athletes do not derive comparable benefits from these medications.

Due to their abuse as stimulants and anabolic agents, excessive doses of terbutaline and salbutamol are prohibited during sporting events [3–6]. Only athletes with asthma or exercise-induced asthma are permitted to use this class of medication via inhalation; the World Anti-Doping Agency (WADA) and the International Olympic Committee (IOC) require athletes to demonstrate medical necessity and obtain authorization for therapeutic use. As a result, beta-2 agonists are subject to a complex set of regulations and are included in the WADA Prohibited Substances List^2^.

Terbutaline, commonly used for the treatment of asthma, chronic obstructive pulmonary disease, cough, and bradyarrhythmia, as well as for tocolytic therapy, is one of the most widely used beta-2 agonists [7–10]. Furthermore, according to Uzunhisarcıklı (2024), terbutaline is a highly effective drug for lung diseases and is considered a potential treatment for lung cancer [7,8]. High doses of terbutaline can cause symptoms such as anxiety, chest tightness, palpitations, pruritus, limb numbness, muscle tremors, nervousness, and tachycardia [11–13]. In addition, it is used as a dietary additive in animal husbandry [14–18]. Since terbutaline overdose is considered doping in sports competitions, its rapid and reliable detection is crucial.

Liquid chromatography–tandem mass spectrometry [11,19,20], ultraperformance liquid chromatography [18,21], microemulsion high-performance liquid chromatography [22], high-performance thin-layer chromatography [23,24], and chemiluminescence are among the analytical techniques developed for the determination of terbutaline hemisulfate (THS) [18]. Although these techniques are effective for the determination of THS, they have several disadvantages, including complex sample preparation procedures, such as liquid–liquid extraction and solid-phase extraction, long analysis times, and the need for expensive instrumentation [25]. In contrast, electrochemical sensors offer several advantages, including rapid response, the elimination of extraction steps, ease of use, and cost-effectiveness [26,27]. One type of electrode commonly used as an electrochemical sensor is the carbon paste electrode (CPE).

CPEs are still widely used for the analysis of organic compounds. CPEs are electrochemical sensors used for the determination of organic compounds that undergo anodic oxidation. The optimal selection of both graphite and the liquid binder is one of the most important factors influencing the performance of carbon pastes. The widespread use of CPEs is attributed to their ease of modification and, consequently, their adaptability in achieving enhanced selectivity and sensitivity for newly developed analytical methods. The advantages of CPEs include a wide potential window, low background current, easy regeneration of the electrode surface, and the possibility of chemical or biological modification. In this study, a nanosensor for the voltammetric determination of THS was developed by modifying a carbon paste electrode with tin oxide (SnO_2_) nanoparticles [28].

Recently, metal oxide nanoparticles have been increasingly used in the fabrication of electrochemical sensors. Owing to their large surface area and unique electrical properties, metal nanoparticles exhibit physicochemical properties that differ from those of their bulk counterparts, making them suitable for applications in biotechnology and biomedicine [29–32]. Among various metal oxides, tin oxide (SnO_2_) nanoparticles have attracted considerable attention because of their unique properties.

SnO_2_ is a unique material with several advantageous properties, including low operating temperature, high thermal stability, and a high degree of transparency in the visible spectrum. Owing to its multiple valence states and the presence of oxygen vacancies, SnO_2_ is regarded as a highly versatile metal oxide. Tin can exist in two oxidation states, +2 and +4, in tin oxides. Its high carrier density, oxygen vacancies, chemical stability, and remarkable electrical, optical, and electrochemical properties make SnO_2_ an n-type semiconductor with a wide band gap of 3.6 eV at 300 K. It has a wide range of applications in solar cells, catalytic support materials, transparent electrodes, and solid-state chemical sensors. Applications such as catalysis, gas sensors, transparent electrodes, and Li-ion batteries particularly benefit from this wide band gap. Because of its high electron mobility (100–200 cm^2^/V·s), photogenerated electrons can be transported more rapidly. SnO_2_ is widely used in smoke sensors, humidity sensors, gas sensors, photocatalysts, hot-end coatings, electrodes, solar collectors, photovoltaic cells, and double-glazed glazing systems. It is also an effective oxidation catalyst [33].

The aim of this study was to develop a voltammetric method for the determination of terbutaline using only SnO_2_ nanoparticles, an n-type semiconductor, without the need for composite materials. According to the literature, the voltammetric determination of terbutaline has predominantly been performed using electrodes containing p-type semiconductors or composite materials. Although n-type nanoparticles such as TiO_2_ and ZrO_2_ have been used for the determination of terbutaline, they have generally been combined with modifiers such as multiwalled carbon nanotubes (MWCNTs) and ionic liquids (ILs). The SnO_2_ nanoparticle-modified carbon paste electrode developed in this study differs from previously reported electrodes in that it enables the determination of terbutaline without the need for additional composite modifying materials. This study aimed to develop a rapid, cost-effective, and simple method for the determination of terbutaline without the need for additional modification or composite materials.

## 2. Materials and methods

### 2.1. Materials

#### 2.1.1. Chemicals

Synthetic graphite powder (1–2 μm; Sigma-Aldrich, St. Louis, MO, USA), mineral oil (Sigma-Aldrich, St. Louis, MO, USA), THS (C_12_H_19_NO_3_·0.5H_2_SO_4_; molecular weight: 274.32 g/mol; Sigma-Aldrich, St. Louis, MO, USA), acetic acid (CH_3_COOH, 100%; density: 1.05 kg/L; Merck, Darmstadt, Germany), sodium hydroxide (Riedel-de Haën, Seelze, Germany), and all other chemicals were purchased from Sigma-Aldrich Chemical Co. (St. Louis, MO, USA). In the experiments, ready-to-use SnO_2_ nanoparticles with a spherical morphology, a purity of 99.87%, and an average particle size of 30–60 nm were obtained from Nanography (Ankara, Türkiye; product code: NG04SO3301) and used without further treatment. For sample analysis, Bricanyl syrup (AstraZeneca, Cambridge, United Kingdom), containing 1.5 mg of terbutaline hemisulfate and 66.5 mg of guaifenesin (guaiacol glyceryl ether) per 5 mL, was used. All solutions used in the experiments were freshly prepared immediately before use. THS solutions were prepared in acetate buffer that had been purged with nitrogen gas for 2 min.

#### 2.1.2. Apparatus

The experiments were performed using a Metrohm Autolab Type 3 potentiostat (Metrohm Autolab, Utrecht, The Netherlands) operated with Nova 1.9 software and a conventional three-electrode system consisting of a carbon paste electrode (glass tube, 5 cm in length and 4 mm in diameter) as the working electrode, an Ag/AgCl electrode as the reference electrode, and a platinum wire as the counter electrode. A MiniFlex II X-ray diffractometer (Rigaku, Tokyo, Japan), a Gemini SEM 560 scanning electron microscope (ZEISS, Oberkochen, Germany), an Ultim Max 40 energy-dispersive X-ray spectroscopy system (Oxford Instruments, Abingdon, United Kingdom), Gilson P100 and P1000 micropipettes (Gilson, Villiers-le-Bel, France), and a Yellow-Line magnetic stirrer (IKA, Staufen, Germany) were also used. High-purity water (18 MΩ·cm) was obtained using a USF ELGA UHQ water purification system (ELGA LabWater, High Wycombe, United Kingdom).

### 2.2. Characterization of SnO_2_ nanoparticles

X-ray diffraction (XRD) analysis was performed to identify the crystalline phases present in the SnO_2_ nanoparticles. The crystal structure of the SnO_2_ nanoparticles was investigated using an X-ray diffractometer (MiniFlex II, Rigaku, Tokyo, Japan). The SnO_2_ nanoparticles were analyzed over a 2θ range of 3°–70° using Cu Kα radiation (λ = 1.5406 Å) operated at 30 kV and 15 mA, with a step size of 0.0200° and a scan rate of 2.0000°/min.

### 2.3. Preparation of the carbon paste electrode

Graphite powder and mineral oil were mixed in appropriate proportions to prepare SnO_2_ nanoparticle-modified carbon paste electrodes (SnO_2_NP/CPE). For this purpose, the SnO_2_NP-modified carbon paste electrode was prepared using two pastes with different compositions: a small paste and a large paste. The small paste was prepared by thoroughly mixing 0.065 g of graphite powder, 0.005 g of SnO_2_ nanoparticles, and 0.03 g of mineral oil until a homogeneous paste was obtained. The large paste was prepared by mixing 0.7 g of graphite powder with 0.3 g of mineral oil until a homogeneous paste was obtained. The small paste was first packed into a cylindrical glass tube (inner diameter: 3.3 mm; length: 4 cm). The remaining space was then filled with the large paste and compacted using a steel rod. Electrical contact with the electrode was established using a copper wire. Unmodified CPEs were also prepared using the same procedure, except that only 0.7 g of graphite powder and 0.3 g of mineral oil were mixed. The surface morphologies of the prepared bare CPE and SnO_2_ nanoparticle-modified CPE were characterized using scanning electron microscopy (SEM), and their elemental compositions were analyzed using energy-dispersive X-ray spectroscopy (EDS).

### 2.4. Electrochemical measurements

Electrochemical measurements were performed in a voltammetric cell using cyclic voltammetry (CV) and differential pulse voltammetry (DPV). Before each voltammetric measurement, the prepared bare CPE and SnO_2_NP/CPE were immersed in a voltammetric cell containing 0.25 M acetate buffer (pH 4.0). After the solution had been purged with nitrogen gas for 3 min, background voltammograms were recorded. The electrochemical determination of THS was performed by adding known concentrations of THS to the voltammetric cell containing the acetate buffer solution. Before each voltammetric measurement, the solution was stirred in the voltammetric cell for 3 min. CVs of both the CPE and SnO_2_NP/CPE were recorded in acetate buffer and THS-containing solutions over the potential range of 0–1.2 V at a scan rate of 10 mV/s. DPVs were recorded in the anodic direction over the potential range of 0–1.2 V under the optimized experimental conditions. The response time of the developed SnO_2_NP/CPE was 2 min. In addition, electrochemical impedance spectroscopy (EIS) measurements were performed using a conventional three-electrode electrochemical cell consisting of a working electrode, an Ag/AgCl reference electrode, and a platinum wire counter electrode. A 0.25 M acetate buffer (pH 4.0) containing 1 mM [Fe(CN)_6_]^3−/4−^ was used as the standard redox probe.

### 2.5. Preparation of the pharmaceutical sample

Sample analysis was performed using commercially available Bricanyl syrup. An aliquot of 0.685 mL of Bricanyl syrup was diluted to 25 mL, and DPVs were recorded to determine the THS content of the Bricanyl syrup. The final THS concentration in the prepared sample solution was 30 μM.

## 3. Results and discussion

### 3.1. Characterization of SnO_2_ nanoparticles

**Figure 1:**
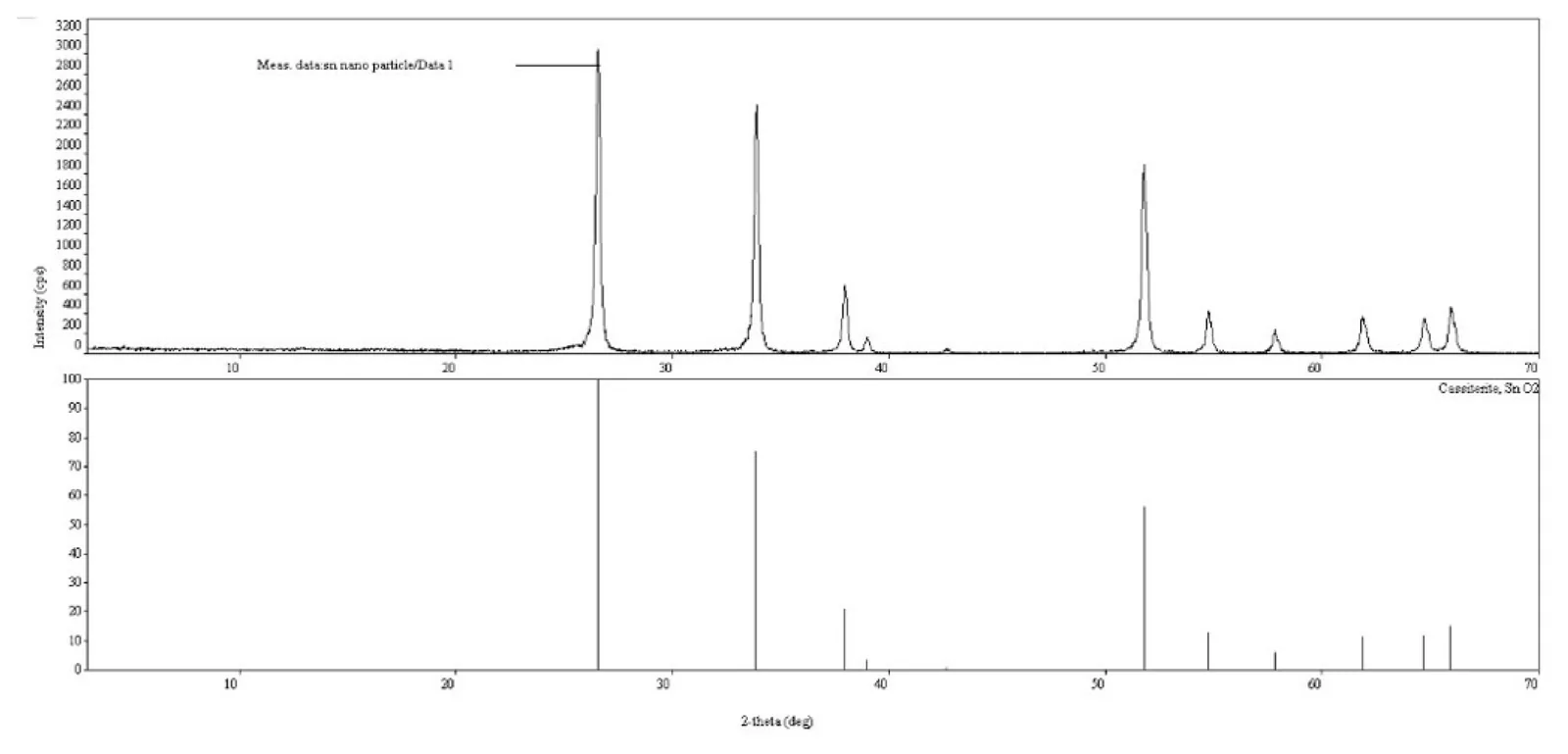
XRD pattern of the SnO_2_ nanoparticles used in this study.

The XRD pattern of the SnO_2_ nanoparticles used in the experiments is shown in Figure 1. All diffraction peaks were indexed with reference to the tetragonal crystal structure of SnO_2_ (JCPDS card no: 41–1445). Strong diffraction peaks observed at 26.56°, 33.88°, 37.90°, 51.80°, 55.90°, and 62.10° correspond to the (110), (101), (200), (211), (220), and (310) crystal planes, respectively, and are in good agreement with the tetragonal crystal structure of SnO_2_.

**Figure 2:**
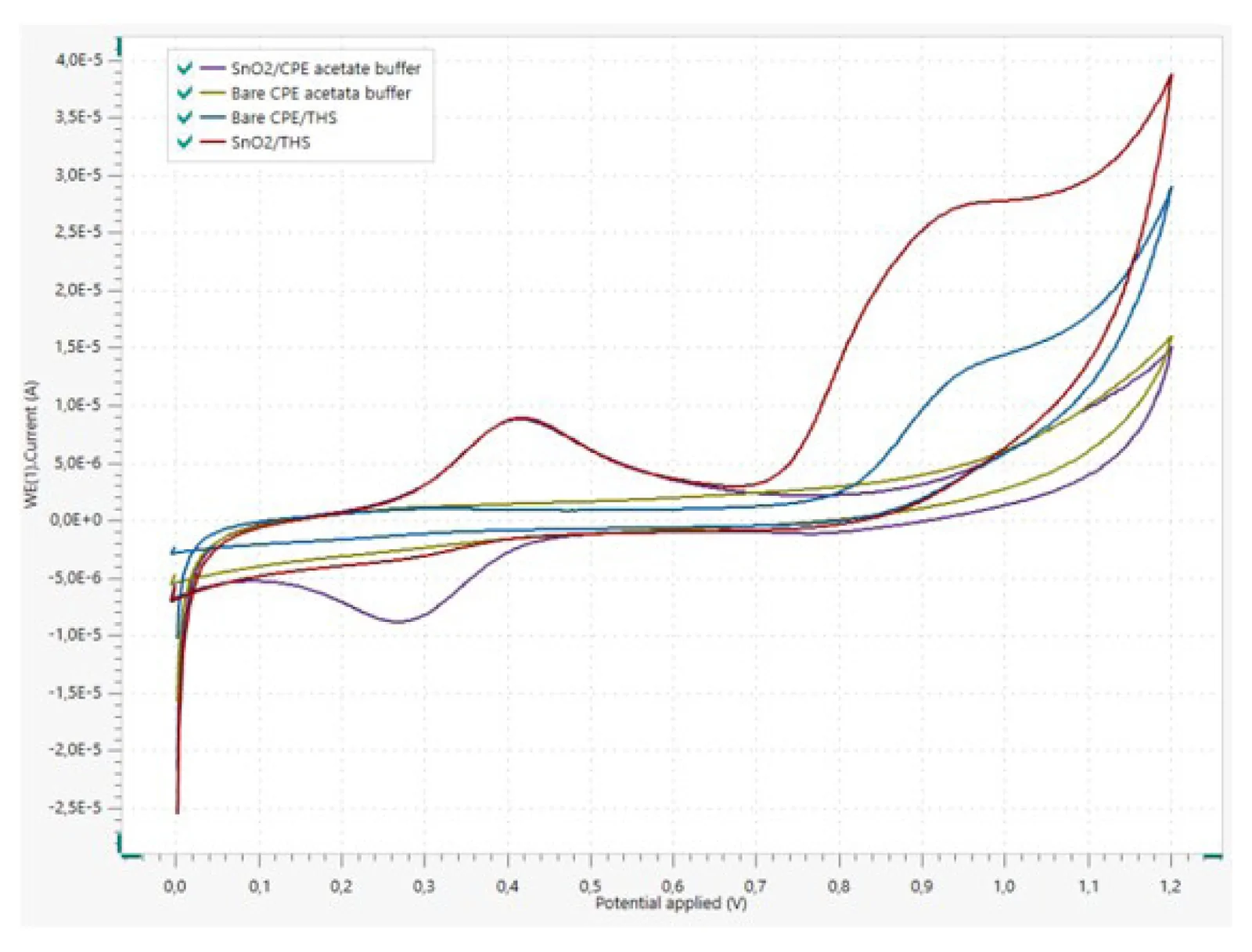
SEM micrographs and EDS spectra of the bare carbon paste (A) and the SnO_2_-modified carbon paste electrode surface (B).

Scanning electron microscopy (SEM) was used to examine the surface morphology of the fabricated electrodes. Figure 2 presents SEM micrographs of the bare carbon paste electrode (Figure 2A) and the SnO_2_ nanoparticle-modified carbon paste electrode (Figure 2B). The EDS elemental analysis results for both electrodes are presented below the corresponding SEM micrographs. The results clearly demonstrate the differences in surface composition before and after modification with SnO_2_ nanoparticles. According to the EDS results, a substantial amount of Sn was detected on the surface of the SnO_2_NP/CPE, confirming the successful modification of the electrode.

### 3.2. Electrochemical behavior of the SnO_2_NP/CPE

**Figure 3:**
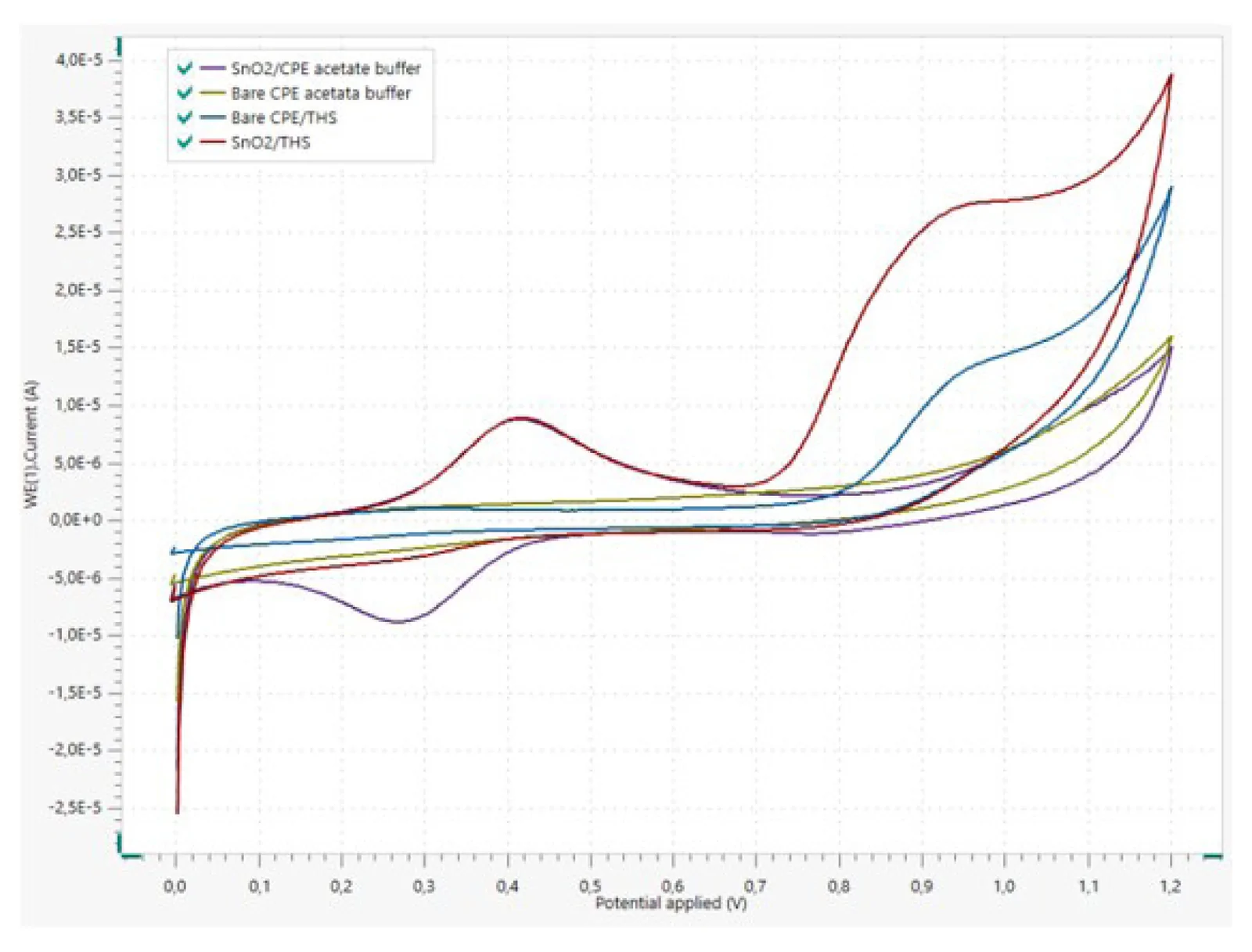
Cyclic voltammograms of the bare CPE and SnO_2_NP/CPE recorded in 0.25 M acetate buffer (pH 4.0) containing 1 mM THS (SnO_2_ content: 5%(w/w), scan rate: 10 mV/s, temperature: 25 °C).

As shown in Figure 3, no peak was observed in the CV of the bare CPE recorded in 0.25 M acetate buffer (pH 4.0). In contrast, the CV recorded under the same conditions using the SnO_2_NP/CPE exhibited two peaks, one anodic and one cathodic. An anodic peak current of approximately 6 μA was observed at 0.42 V, whereas a cathodic peak current of approximately 5 μA was observed at 0.28 V. The peaks observed under these conditions can be identified as oxidation and reduction peaks belonging to the Sn^2+^/Sn^4+^ redox pair.

For the bare CPE, an anodic peak current of 6.7 μA was observed at 0.95 V in 0.25 M acetate buffer (pH 4.0) containing 1.0 mM THS. In the CV recorded using the SnO_2_NP/CPE in the presence of THS, two anodic peaks were observed. One of these peaks appeared at 0.42 V, corresponding to the peak observed in acetate buffer alone, and exhibited a similar peak current. The second anodic peak was observed at approximately 0.9 V, with a peak current of approximately 10 μA. This peak, observed at approximately 0.9 V for both the bare CPE and the SnO_2_NP/CPE, was assigned to the oxidation of THS. The higher peak current observed for the SnO_2_NP/CPE compared with the bare CPE indicates the electrocatalytic effect of the SnO_2_ nanoparticles.

**Figure 4:**
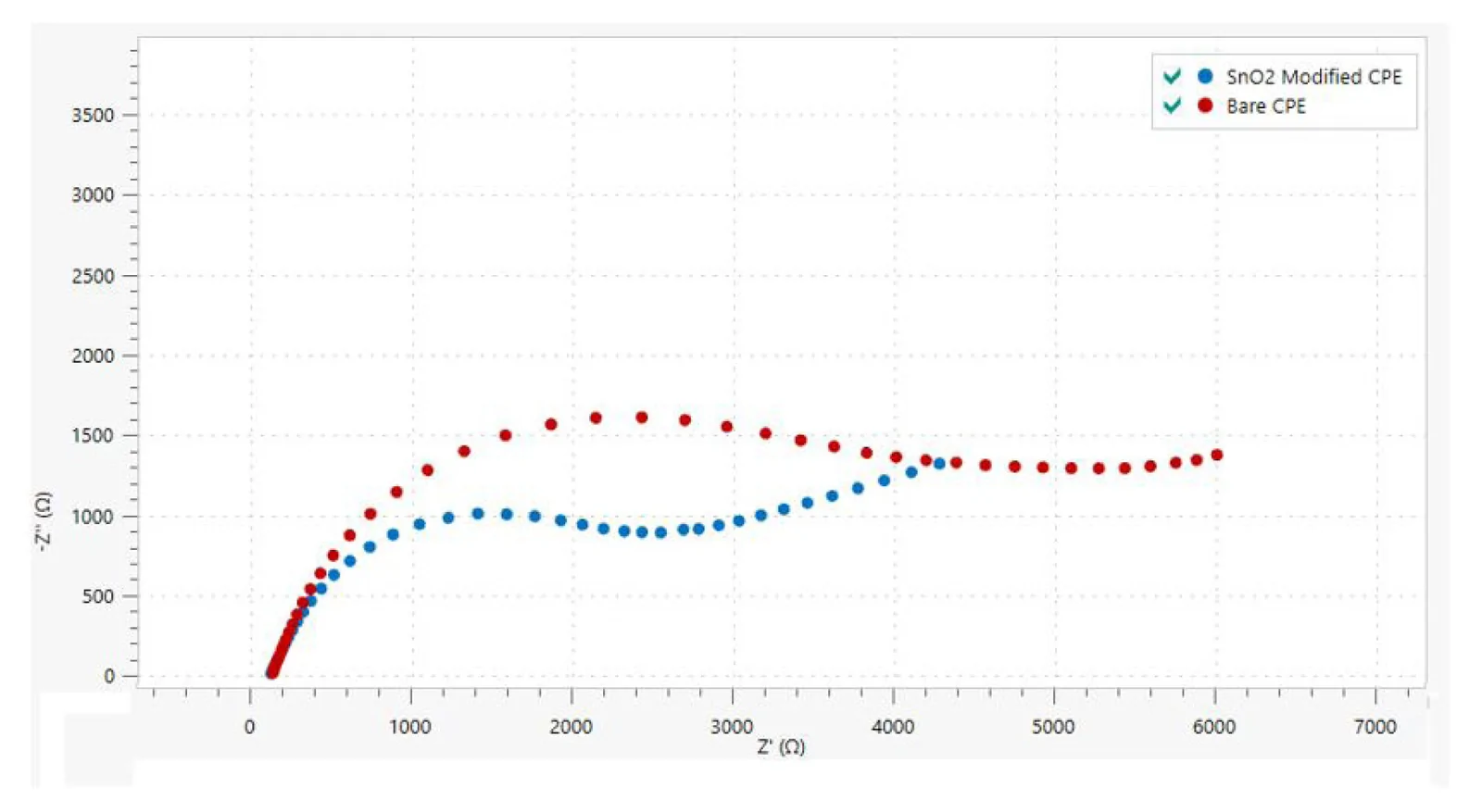
Electrochemical impedance spectra recorded in 0.25 M acetate buffer (pH 4.0) containing 1 mM [Fe(CN)_6_]^3−/4−^

The EIS results for the bare CPE and the SnO_2_ nanoparticle-modified CPE are shown in Figure 4. The bare CPE exhibited a larger semicircle, indicating a higher charge-transfer resistance (R_ct_ = 6.82 kΩ), which suggests slower electron-transfer kinetics. In contrast, the SnO_2_ nanoparticle-modified CPE exhibited a lower charge-transfer resistance (R_ct_ = 2.13 kΩ), indicating more facile electron transfer at the electrode surface. These results suggest that modification with SnO_2_ nanoparticles facilitated electron transfer and enhanced the electrical conductivity of the electrode surface.

### 3.3. Effect of scan rate

**Figure 5:**
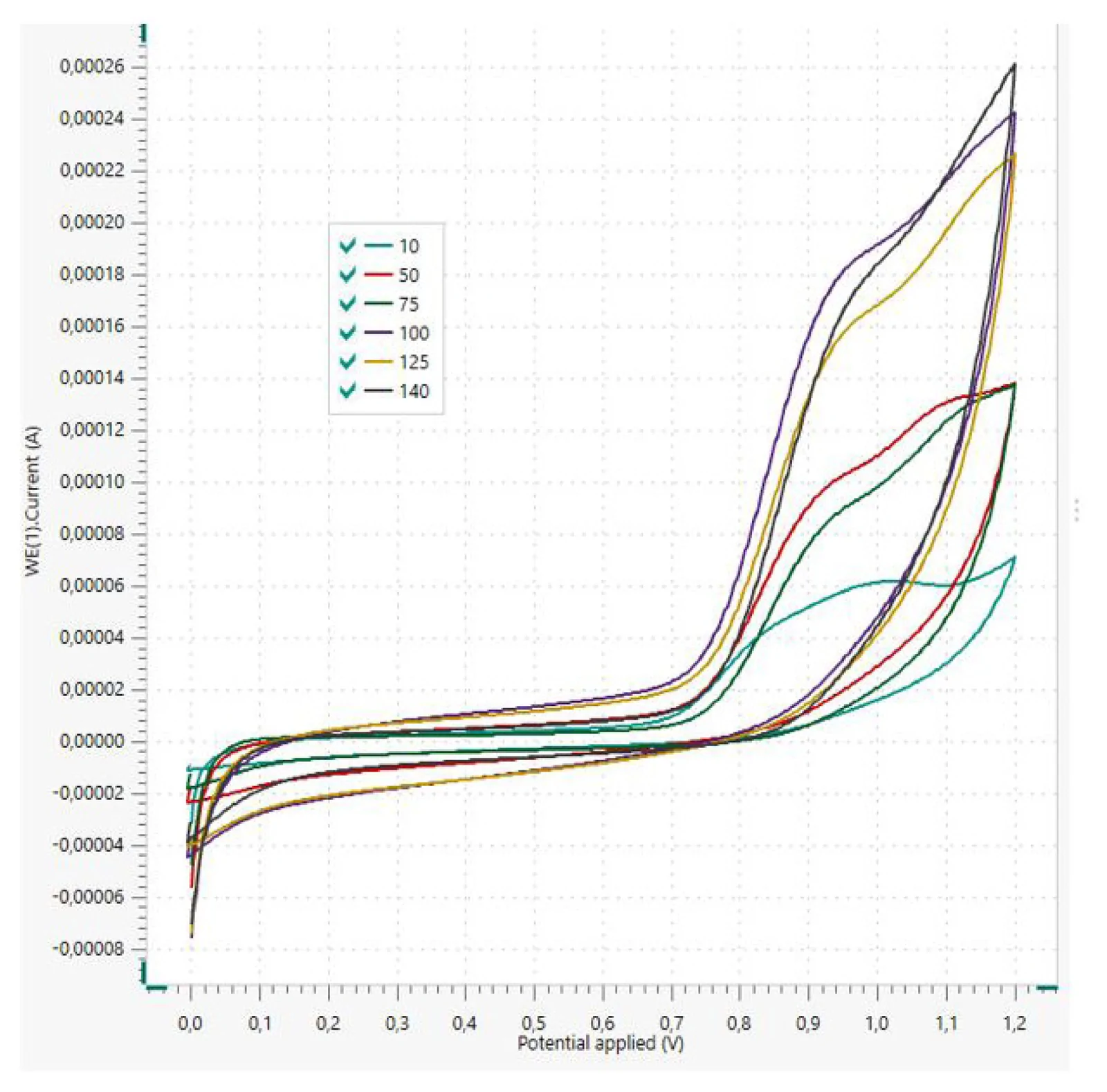
Cyclic voltammograms recorded at scan rates of 10, 50, 75, 100, 125, and 140 mV/s in 0.25 M acetate buffer (pH 4.0) containing 1.0 mM THS (SnO_2_ content: 5%(w/w), temperature: 25 °C).

The effect of scan rate on the peak current for the voltammetric determination of 1.0 mM THS using the SnO_2_NP/CPE was investigated by CV at scan rates of 10, 50, 75, 100, 125, and 140 mV/s. As shown in Figure 5, the peak current increased with increasing scan rate. The value of αn was calculated to estimate the number of electrons involved in the electrochemical process. The Tafel plot was constructed using the following equation:


$$Epa=\left(\frac{2,303RT}{\propto nF}\right)\left(\frac{1}{2}\right)\text{log }v+constant$$

**Figure 6:**
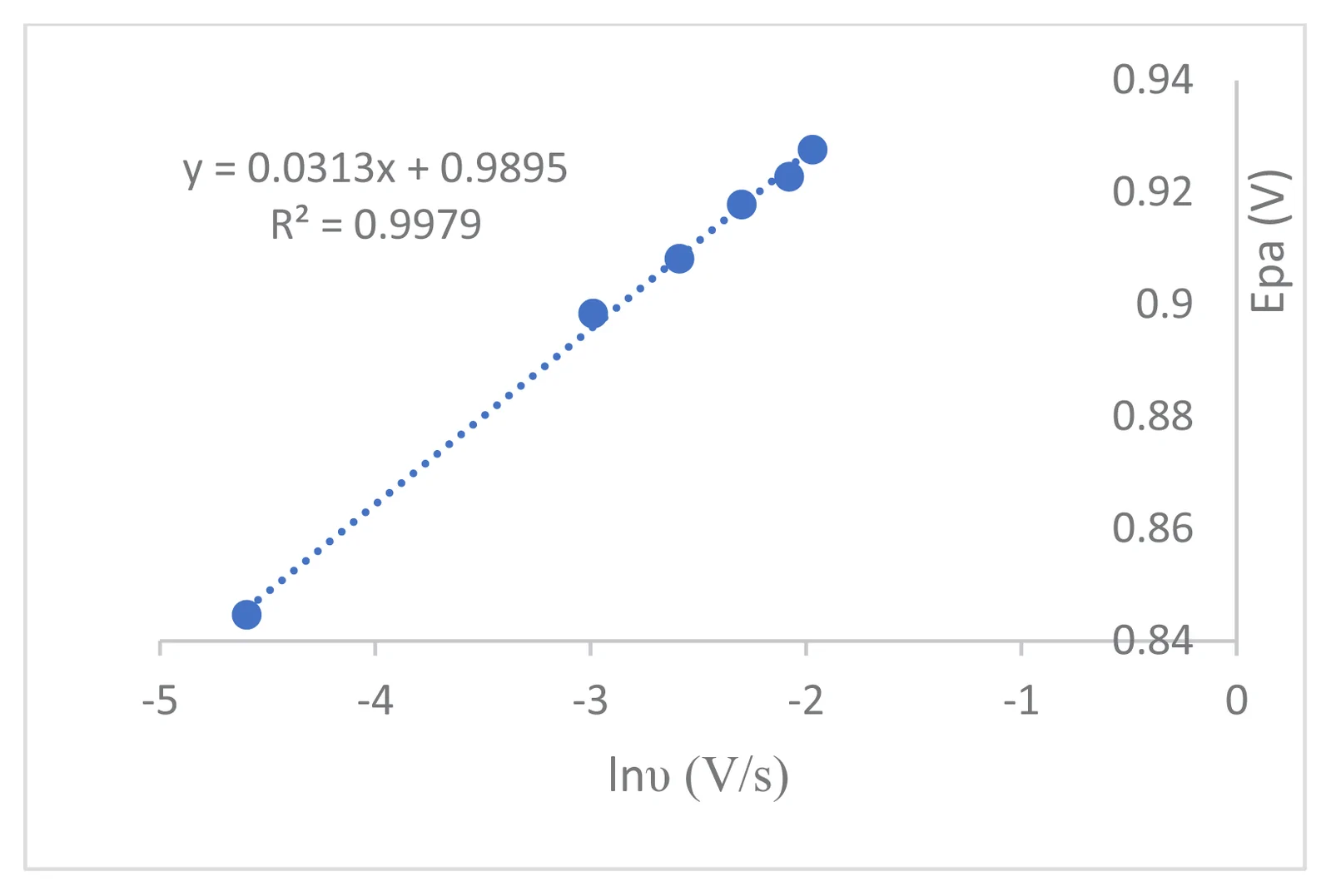
Relationship between the peak potential and lnυ at different scan rates in 0.25 M acetate buffer (pH 4.0) containing 1.0 mM THS (SnO_2_ content: 5%(w/w), temperature: 25 °C).

Figure 6 shows that the oxidation peak potential of THS was proportional to the logarithm of the scan rate, with a slope of 0.0313. Based on these results, and assuming an electron-transfer coefficient (α) of approximately 0.5 for an irreversible process, THS was estimated to undergo a two-electron transfer process [34].

**Figure 7:**
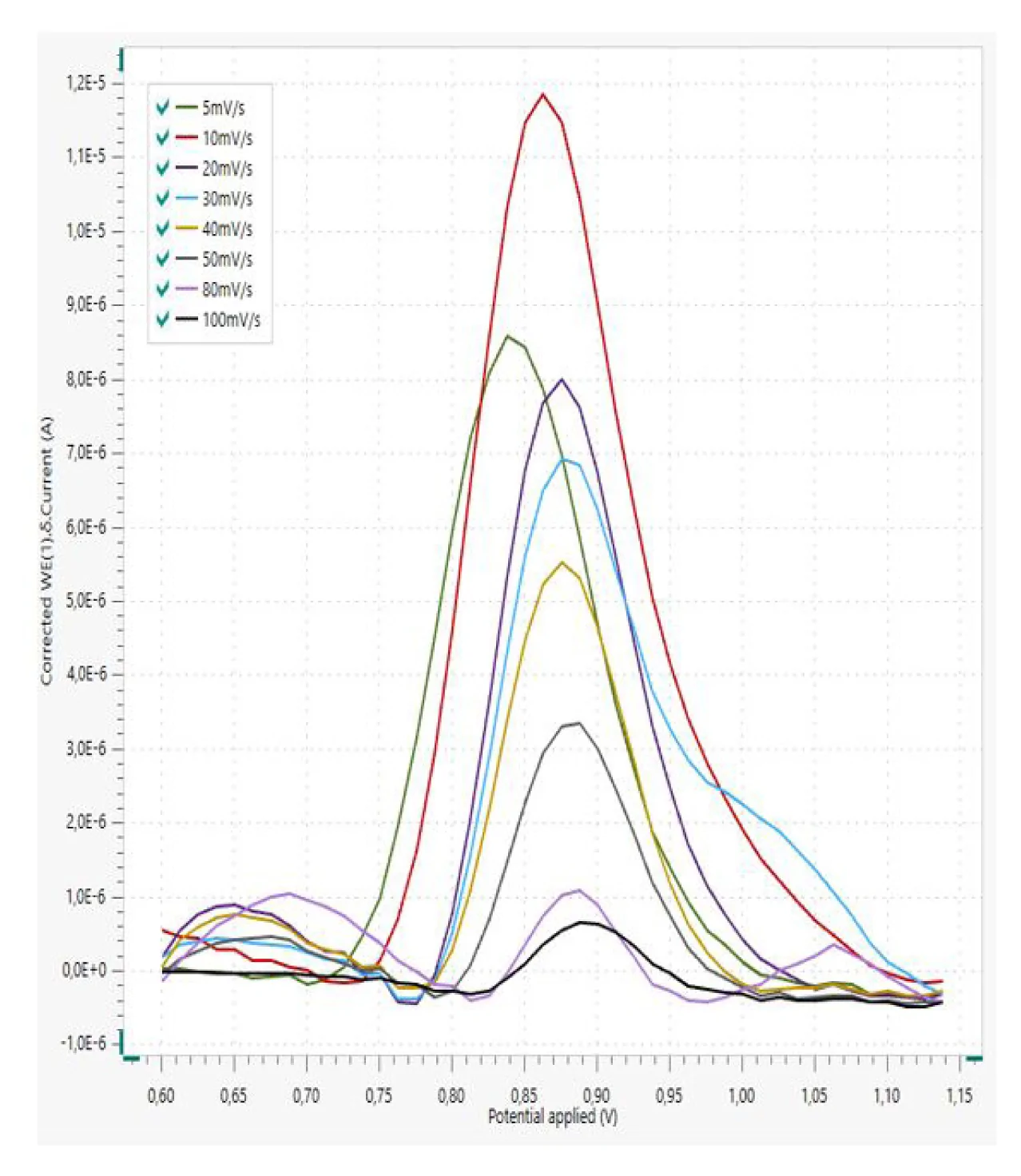
Differential pulse voltammograms recorded at scan rates of 5, 10, 20, 30, 40, 50, 80, and 100 mV/s in 0.25 M acetate buffer (pH 4.0) containing 120.0 μM THS (SnO_2_ content: %5(w/w), temperature: 25 °C).

DPVs were recorded over the potential range of 0–1.2 V at scan rates of 5, 10, 20, 30, 40, 50, 80, and 100 mV/s in 0.25 M acetate buffer (pH 4.0) containing 120.0 μM THS (Figure 7).

**Figure 8:**
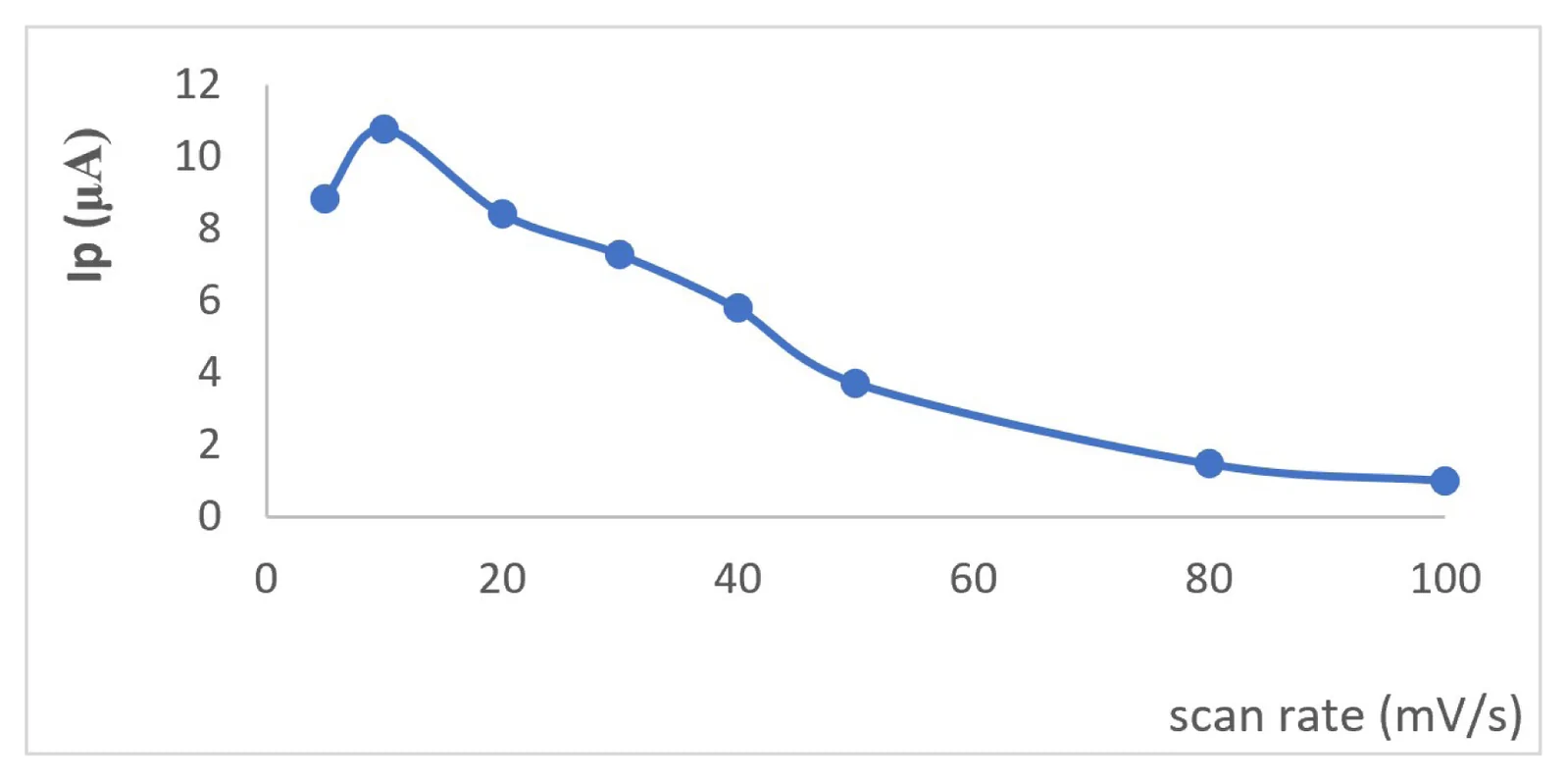
Relationship between scan rate and peak current in 0.25 M acetate buffer (pH 4.0) containing 120.0 μM THS (SnO_2_ content: %5(w/w), temperature: 25 °C).

As shown in Figure 8, the highest peak current was obtained at a scan rate of 10 mV/s. Accordingly, a scan rate of 10 mV/s was used in all subsequent experiments.

In electrochemical sensors, the scan rate directly affects the current response, sensitivity, and the electrochemical processes occurring at the electrode surface. At high scan rates, ion transport at the electrode/electrolyte interface becomes more limited. As a result, the peak potential may shift toward either more positive or more negative values.

### 3.4. Optimization of the SnO_2_ content

**Figure 9:**
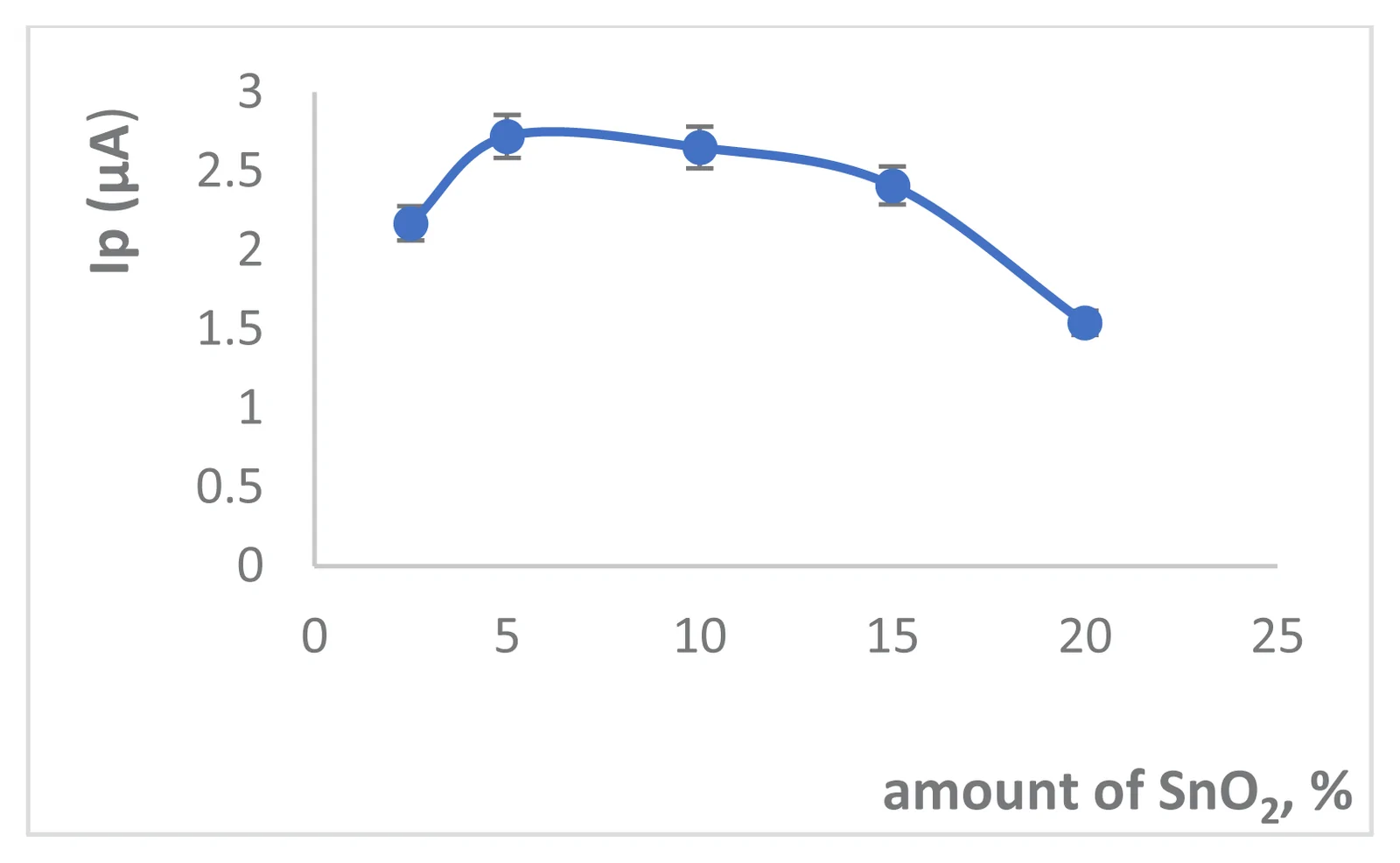
Effect of SnO_2_ content on the peak current in 0.25 M acetate buffer (pH 4.0) containing 30 μM THS (scan rate: 10 mV/s, temperature: 25 °C).

To optimize the SnO_2_ content of the modified electrode, electrodes containing 2.5%, 5%, 10%, 15%, and 20% (w/w) SnO_2_ were prepared. Measurements were performed in a solution containing 30.0 μM THS. Three replicate measurements were performed for each electrode. The variation in peak current values with respect to nanoparticle content is presented in Figure 9. The peak current increased with increasing SnO_2_ content up to 5% (w/w), after which a slight decrease in peak currents was observed. Since the electrode containing 20% (w/w) SnO_2_ showed surface deformation associated with lower graphite content, the electrode containing 5% (w/w) SnO_2_ was used for subsequent experiments.

### 3.5. Effect of pH on the voltammetric response of the SnO_2_NP/CPE

**Figure 10:**
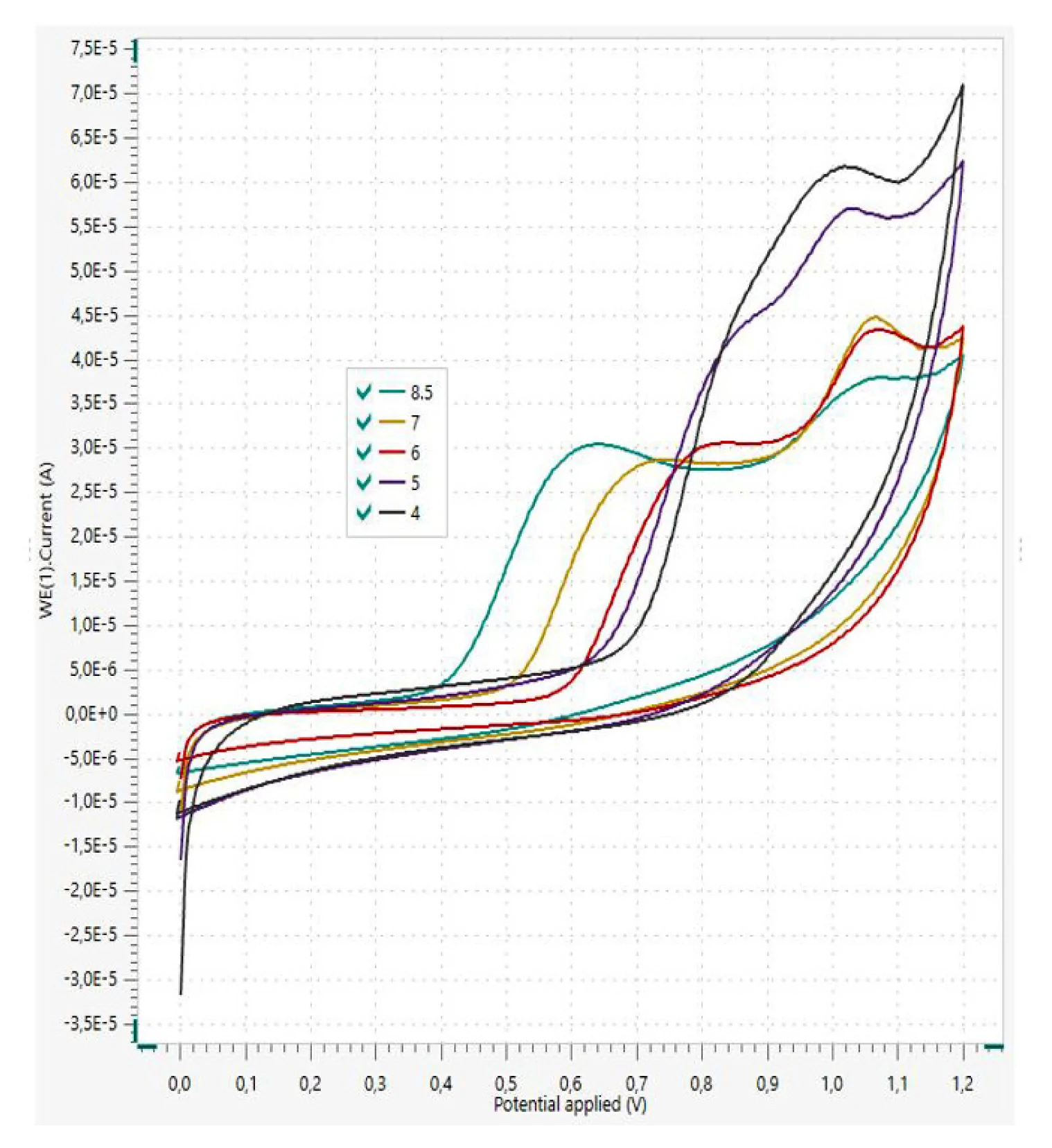
Cyclic voltammograms recorded at different pH values (pH values: 4.0, 5.0, 6.0, 7.0 and 8.5, THS concentration: 1mM, SnO_2_ content: 5%(w/w), temperature: 25 °C).

**Figure 11:**
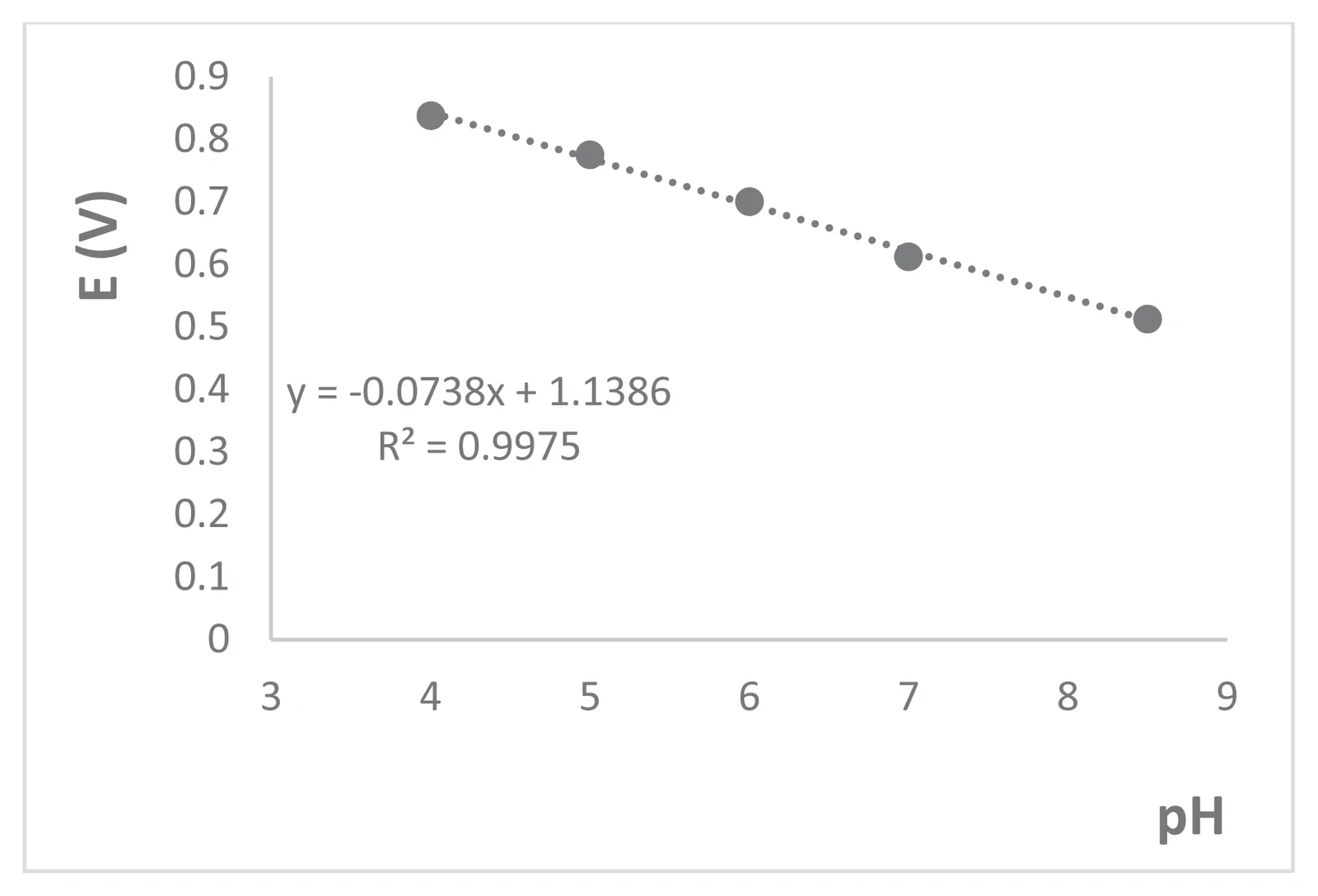
Relationship between peak potential and pH for THS at the SnO_2_NP/CPE (THS concentration: 1.0 mM, SnO_2_ content: 5%(w/w), temperature: 25 °C).

The effect of pH on the electrochemical response of THS was investigated using CV and DPV. To investigate the effect of pH, CVs were recorded in buffer solutions with different pH values containing 1.0 mM THS in the potential range of 0–1.2 V at a scan rate of 10 mV/s (Figure 10). Figure 10 illustrates the shifts in peak potential observed at different pH values. The relationship between peak potential and pH is presented in Figure 11. The results indicate that the oxidation of THS at the SnO_2_NP/CPE is accompanied by proton transfer, resulting in a pH-dependent shift in the oxidation peak potential. As shown in Figure 11, the highest peak current and the most well-defined voltammetric response were observed at pH 4.0. Therefore, pH 4.0 was chosen for the determination of THS. The slope of the Ep–pH plot (73.8 mV/pH) suggests that equal numbers of protons and electrons participate in the oxidation of THS. The experimental results suggest that the oxidation process involves the transfer of two electrons and two protons [34]. The proposed oxidation mechanism of THS is presented in Scheme. The proposed oxidation of terbutaline involves the conversion of the phenolic hydroxyl groups into quinone-type carbonyl (C=O) groups. In the proposed mechanism, the first step involves the loss of one proton and one electron from a hydroxyl group, forming a semiquinone intermediate. In the second step, the loss of one proton and one electron results in the formation of the quinone structure.

**Figure 12:**
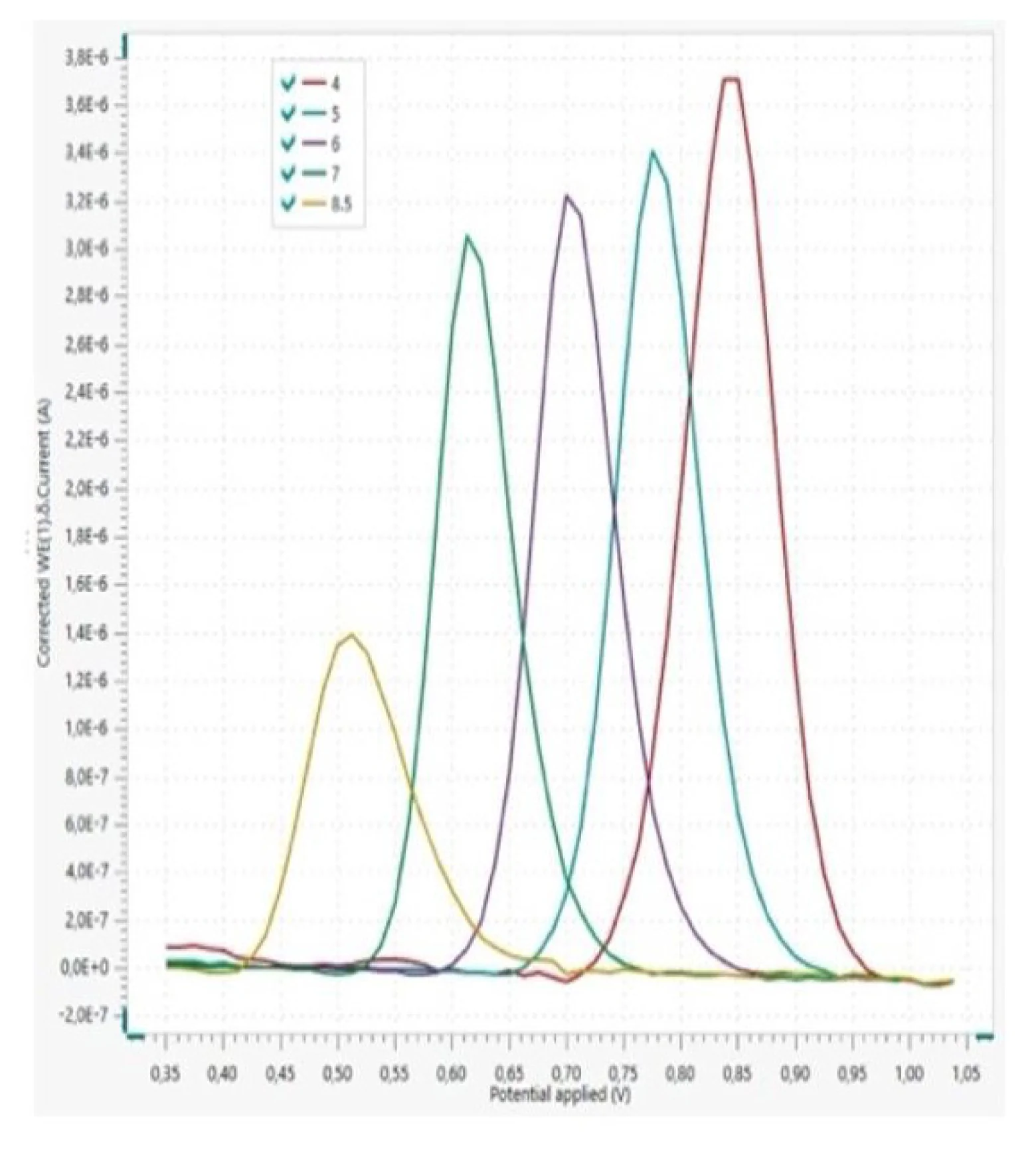
Differential pulse voltammograms recorded at pH 4.0, 5.0, 6.0, 7.0, and 8.5 for 30.0 μM THS (SnO_2_ content: 5%(w/w), temperature: 25 °C).

**Figure 13:**
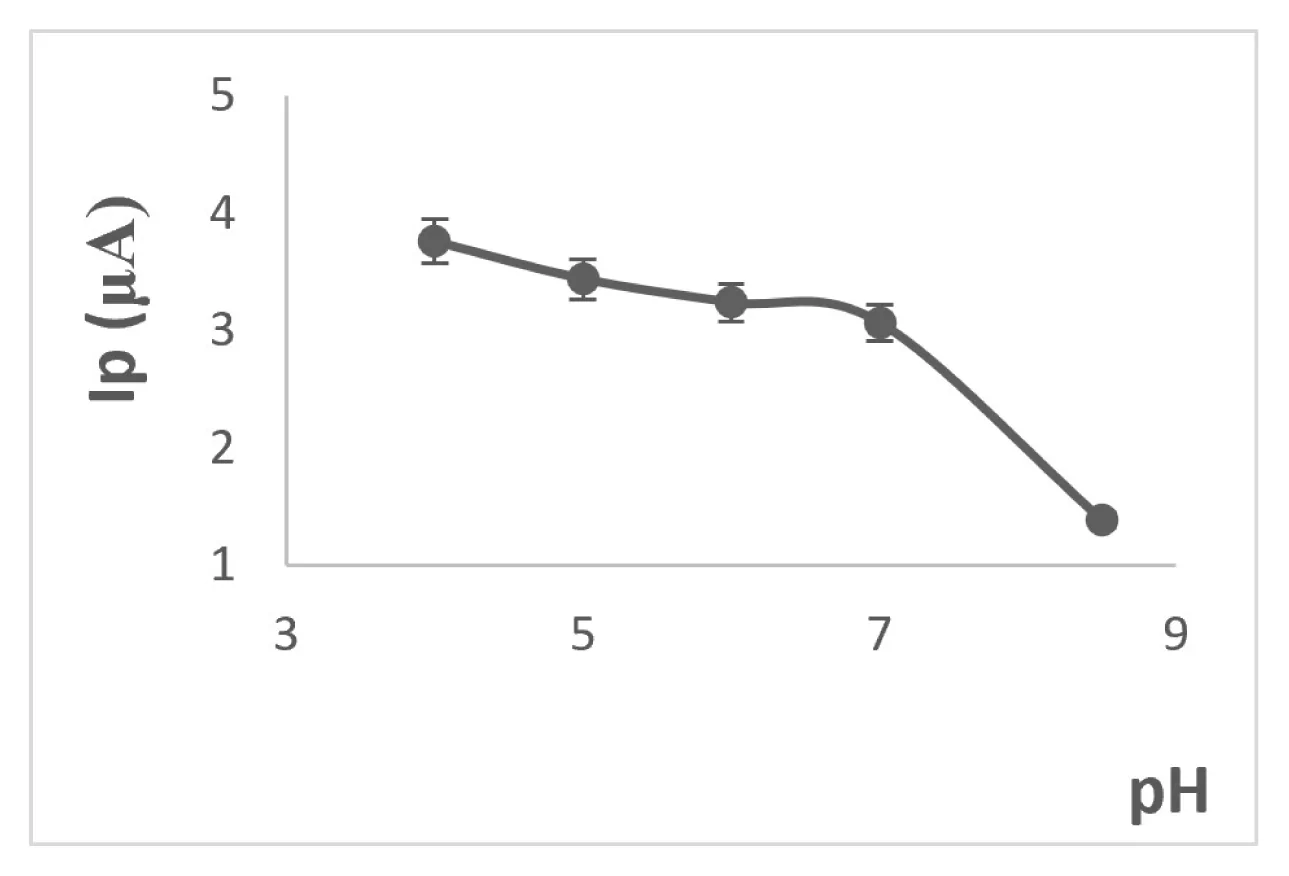
Relationship between peak current and pH for 30.0 μM THS (SnO_2_ content: 5%(w/w), temperature: 25 °C).

Figure 12 shows DPVs recorded at different pH values in 0.25 M acetate buffer containing 30.0 μM THS over the potential range of 0–1.2 V at a scan rate of 10 mV/s. As shown in Figure 13, the highest peak current was obtained at pH 4.0. Accordingly, pH 4.0 was used in all subsequent experiments.

### 3.6. Analytical performance of the SnO_2_NP/CPE

**Figure 14:**
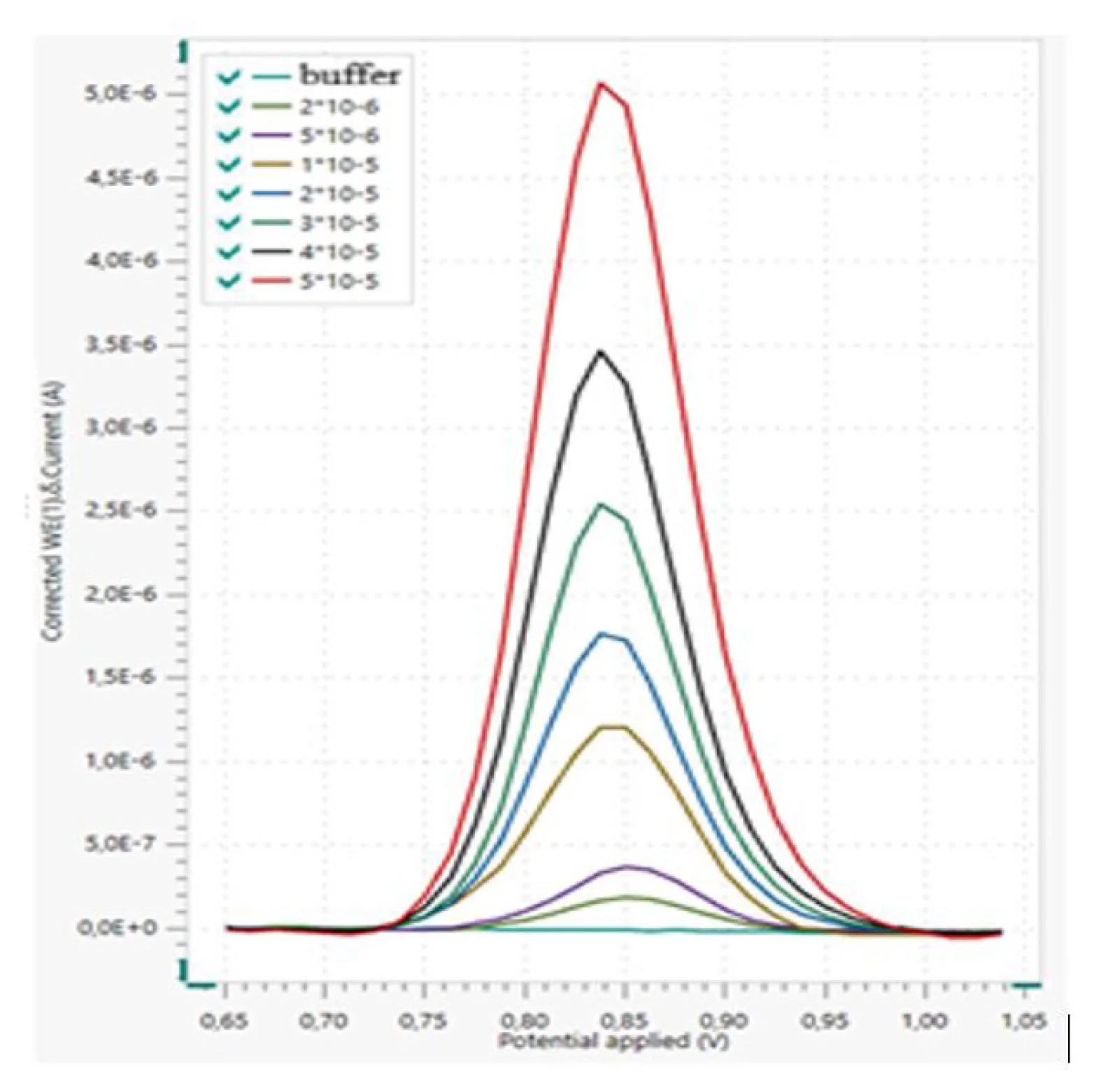
Differential pulse voltammograms recorded at different THS concentrations (2.0–50.0 μM) in 0.25 acetate buffer (pH 4.0) (scan rate: 10 mV/s, SnO_2_ content: 5%(w/w), temperature: 25 °C).

**Figure 15:**
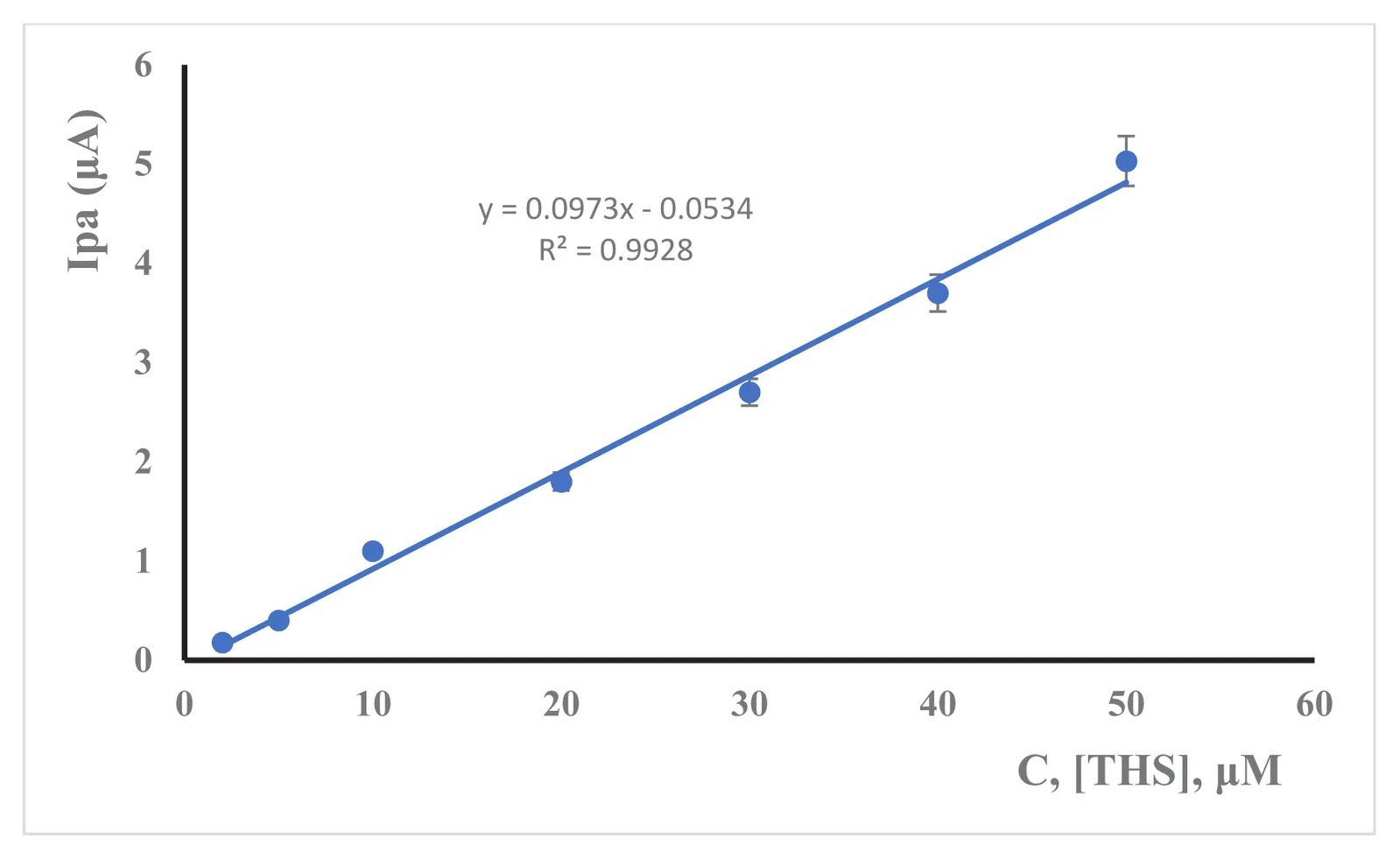
Calibration curve for THS over the concentration range of 2.0–50.0 μM (scan rate: 10 mV/s, 0.25 M acetate buffer (pH 4.0), SnO_2_ content: 5%(w/w), temperature: 25 °C).

Figure 14 shows the DPVs recorded using the SnO_2_NP/CPE at different THS concentrations. Figure 15 shows the linear relationship between the peak current and THS concentration over the range of 2.0–50.0 μM. The calibration curve was constructed using the mean value of three replicate measurements at each concentration. The limit of detection (LOD) for THS was calculated to be 0.55 μM. The LOD was calculated as 3S_b_/m, where S_b_ is the standard deviation of the blank signal and m is the slope of the calibration curve. The limit of quantification was determined to be 1.80 μM. The calibration equation was Ip (μA) = 0.0973C (μM) – 0.0534, with a correlation coefficient of 0.9928.

The repeatability of the method was evaluated using a 30.0 μM THS solution over 10 consecutive measurements, yielding a relative standard deviation of 3.30% (X̄ = 27.99 μM, SD = 0.92). This result indicates good repeatability of the SnO_2_NP/CPE. In addition, reproducibility was evaluated using 10 different SnO_2_NP/CPEs in a 30.0 μM THS solution. The coefficient of variation was 2.58% (X̄ = 28.97 μM, SD = 0.75).

Stability was evaluated by recording measurements at predetermined intervals over 14 days using the prepared electrode in a 30.0 μM THS solution. After 14 days, the peak current remained essentially unchanged compared with its initial value. The absence of a significant decrease in peak current over 14 days indicates good storage stability of the SnO_2_NP/CPE. The developed nanosensor was stored at room temperature when not in use.

As shown in Table 1, modified glassy carbon electrodes (GCEs) have been the most commonly used working electrodes for the electrochemical determination of terbutaline. A comparison of the analytical performance of the proposed sensor with those reported in previous studies is presented in Table 1.

The SnO_2_NP/CPE developed in this study exhibited a wider linear working range than the GN-MWNTs/GC [36], TiO_2_-MWCNTs-IL-GCE [37], MWCNTs/graphene/GCE [38], and poly-EBT/GCE [43]. Its linear working range was comparable to that of the Pt-Pd NPs/COOH-Gr/MoS_2_/GCE [41]. The developed nanosensor has limited applicability for the determination of terbutaline at very low concentrations (pM and nM levels). However, it is well suited for practical and routine analytical applications. In terms of the LOD, the developed SnO_2_NP/CPE exhibited a lower LOD than the bare GCE [44] and an LOD comparable to those of the MWCNTs/graphene/GCE [38], fMWCNTs/CD-R [27], and MWNTs/GCE [45]. A major advantage of the SnO_2_NP/CPE is its low cost and ease of fabrication. In addition, the modification procedure is simpler than those required for more complex nanocomposite-based electrodes. The proposed method also provides a short analysis time for terbutaline determination. Furthermore, stability data for many of the electrodes listed in Table 1 are unavailable. In contrast, the SnO_2_NP-modified CPE retained its initial current response after 14 days, suggesting good storage stability and potential suitability for long-term use in terbutaline determination.

### 3.7. Interference study

**Figure 16:**
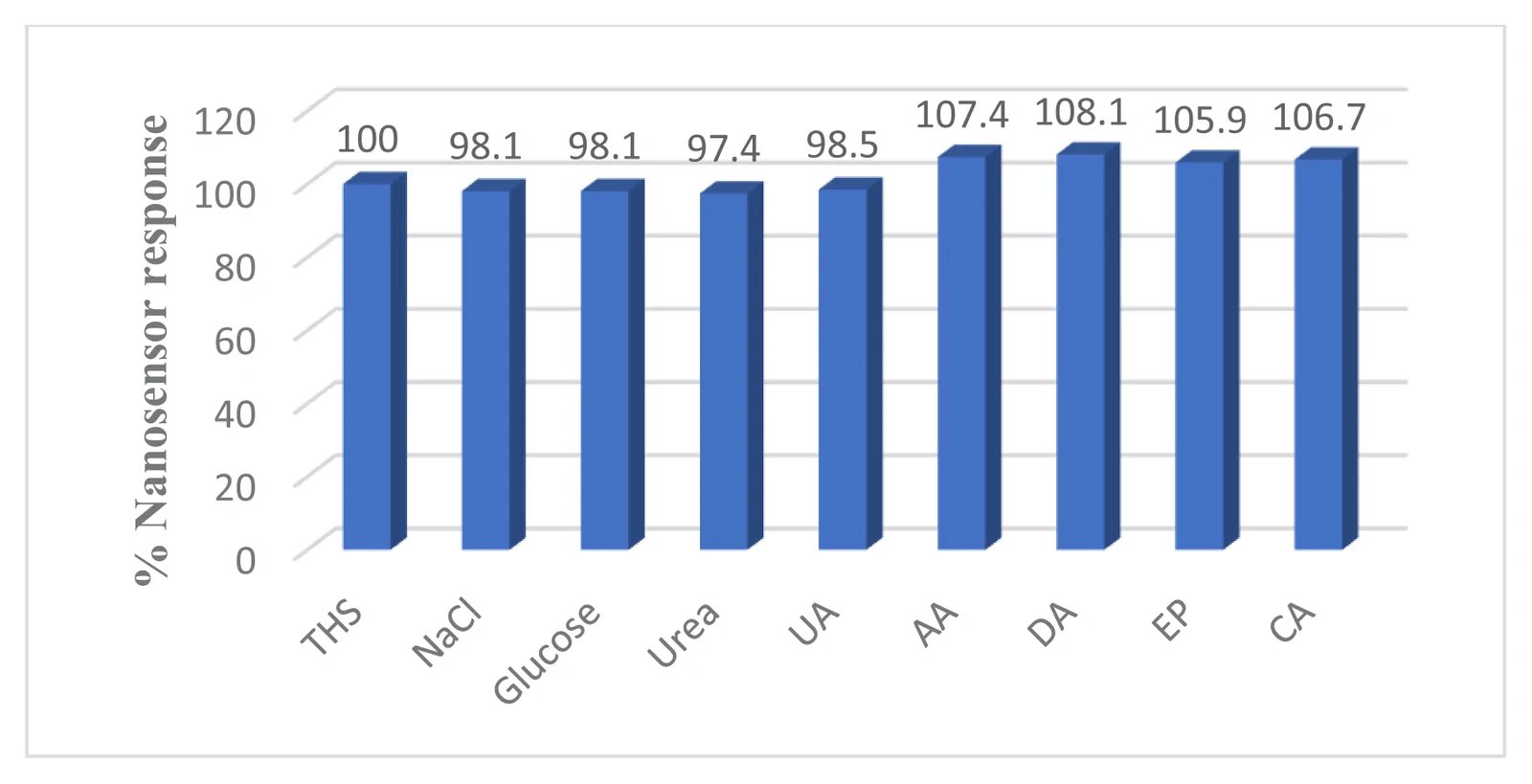
Effect of potential interferents on the voltammetric response of the SnO_2_NP/CPE (0.25 M acetate buffer (pH 4.0), scan rate: 10 mV/s, SnO_2_ content: 5%(w/w), temperature: 25 °C).

To evaluate potential interference effects, DPVs were recorded in 0.25 M acetate buffer (pH 4.0) containing 30.0 μM THS in the presence of 30.0 μM sodium chloride (NaCl), glucose, urea, dopamine (DA), citric acid (CA), epinephrine (EP), ascorbic acid (AA), or uric acid (UA). The interference effect was expressed as the percentage nanosensor response relative to the initial peak current of the THS (Figure 16). No significant interference was observed in the presence of NaCl, glucose, urea, or UA, even when these potential interferents were present at concentrations 10-fold higher than that of THS. In contrast, AA, DA, EP, and CA produced a slight positive interference effect. The interference effects of AA, DA, EP, and CA increased when these compounds were present at 10-fold higher concentrations. The molecular structures of AA, DA, and EP contain electroactive functional groups that can undergo oxidation during the anodic scan, leading to the formation of quinone-type products. Oxidation of these compounds at potentials close to that of THS may result in peak overlap, signal suppression, and distortion of the current response, thereby causing analytical interference.

### 3.8. Analysis of the pharmaceutical sample

The proposed sensor was applied to the determination of THS in Bricanyl syrup (1.5 mg THS per 5 mL). A 0.685 mL aliquot of Bricanyl syrup was diluted to 25 mL with 0.25 M acetate buffer (pH 4.0) to obtain a final THS concentration of 30.0 μM. The prepared sample was analyzed in triplicate. THS concentrations were calculated from the calibration curve using the measured peak currents, and the mean concentration and standard deviation were determined. The results are presented in Table 2. The proposed sensor enabled the sensitive determination of THS in Bricanyl syrup.

## 4. Conclusion

Voltammetric determination of THS using the SnO_2_NP/CPE was achieved over a wide linear concentration range with a low LOD. THS was successfully determined in Bricanyl syrup using the analytically characterized SnO_2_NP/CPE. The proposed sensor is easy to fabricate and cost-effective. Furthermore, the peak current remained essentially unchanged after 14 days compared with its initial value, indicating good storage stability. The SnO_2_NP/CPE also exhibited good repeatability and reproducibility. In conclusion, the SnO_2_NP/CPE represents a promising alternative platform for the determination of terbutaline because of its rapid, cost-effective, and simple fabrication, wide linear concentration range, and ability to accurately determine terbutaline in the presence of potential interferents.

## Figures and Tables

**Scheme:**
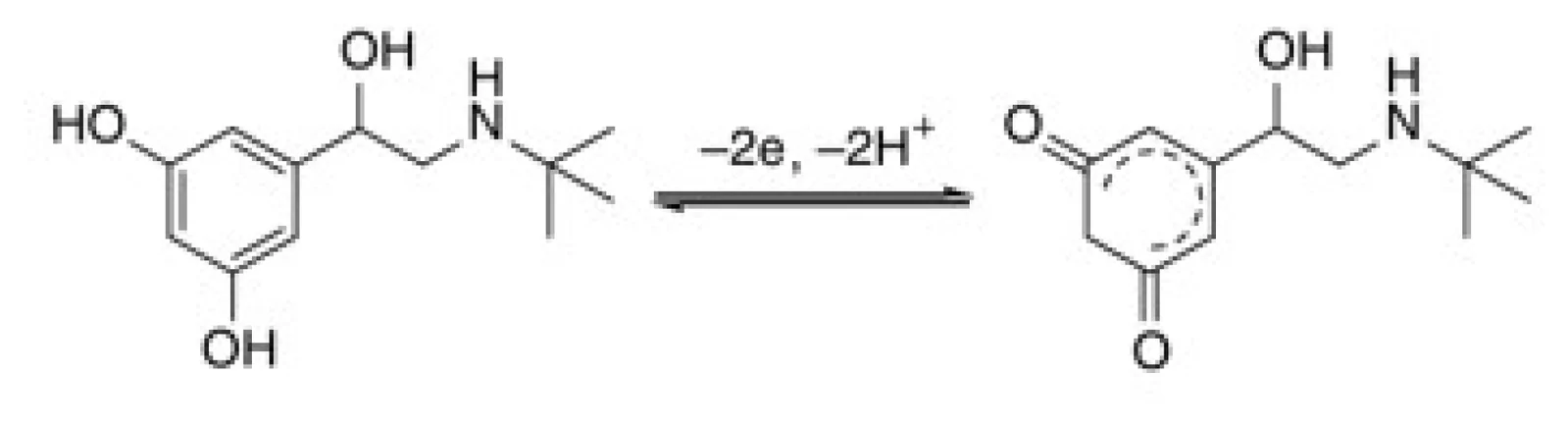
Proposed electrochemical oxidation mechanism of THS.

**Table 1:** Comparison of the analytical performance of the SnO_2_NP/CPE with previously reported electrodes for terbutaline determination.

Electrode	Method*	Linear range (μM)	LOD (μM)	Reference
RGO/GCE	DP-ASV	0.08–34.0	0.0052	[35]
GN-MWNTs/GC	CV	0.2–5.0 and 5.0–40	-	[36]
TiO_2_-MWCNTs-IL-GCE	DPV	0.5–3.0	0.016	[37]
GNS-PWCNT-PANI	AS-DPV	0.02–80.0	0.00583	[12]
MWCNTs/graphene/GCE	DPV	2.0–40.0	0.6527	[38]
Poly(L-arginine)/graphene Nafion/GCE	LSV	0.04–5.0	0.012	[39]
Poly(L-arginine)/GCE	SWV	0.10–22	0.10	[40]
Pt-Pd NPs/COOH-Gr/MoS_2_/GCE	DPV	0.47–14.2 and 0.55–14.92	0.18 and 0.44	[41]
fMWCNTs/CD-R	FIA	3.0–500	0.58	[27]
Er_2_O_3_@GRNP/GCE	SWV	0.0012–0.71	0.000685	[42]
Poly-EBT/GCE	DPV	0.12–2.0	0.015	[43]
ZrO_2_NPs/MWCNTs/GCE	SWV	0.010–0.160	0.00225	[34]
GCE	SWV	8.0–800	6.0	[44]
MWNTs/GCE	DPV	1.12–141	0.34	[45]
SnO_2_NP/CPE	DPV	2.0–50.0	0.55	This study

* *Abbreviations:* DP-ASV: Differential pulse anodic stripping voltammetry, AS-DPV: Anodic stripping – differential pulse voltammetry, LSV: Linear sweep voltammetry, SWV: Square wave voltammetry, FIA: Flow injection analysis.

**Table 2:** Determination of THS in Bricanyl syrup using the SnO_2_NP/CPE.

	Bricanyl syrup ingredient (μM)	Nanosensor response (μM)
Sample analysis	30 μM	29.02 ± 0.47

## Footnotes

1 Hsu E, Bajaj T (2025). Beta2-Agonists. In: StatPearls [Online]. Treasure Island (FL): StatPearls Publishing Website https://www.ncbi.nlm.nih.gov/books/NBK542249/ [accessed 30 July 2024].

2 Prohibited list-2024. World Anti Doping Agency. [Online]. Website www.wada-ama.org [accessed 30 July 2024].
